# Supplementary material for: Evaluation of global outbreak surveillance performance for high pathogenicity avian influenza and African swine fever
Source: Nat Commun. 2025 May 21;16:4737. doi: 10.1038/s41467-025-60094-9 (PMC12095688; doi:10.1038/s41467-025-60094-9)
Supplement: Supplementary file 1 — Supplementary Information [file 41467_2025_60094_MOESM1_ESM.pdf]

Supplementary Information for

Evaluation of Global Outbreak Surveillance Performance for High Pathogenicity Avian Influenza and African Swine Fever

Younjung Kim[1,2]*, Guillaume Fournié[3,4,5], Paolo Tizzani[6], Gregorio Torres[6], Raphaëlle Métras[1], Dirk Pfeiffer[3,7], Pierre Nouvellet[2]

1 Sorbonne Université, INSERM, Institut Pierre Louis d'Épidémiologie et de Santé Publique (IPLESP), UMRS 1136, Paris, France

2 Department of Ecology and Evolution, School of Life Sciences, University of Sussex, Brighton and Hove, UK

3 Department of Pathobiology and Population Sciences, Royal Veterinary College, London, UK

4 Université de Lyon, INRAE, VetAgro Sup, UMR EPIA, Marcy l'Etoile, France

5 Université Clermont Auvergne, INRAE, VetAgro Sup, UMR EPIA, Saint-Gènes-Champanelle, France

6 World Organisation for Animal Health, Paris, France

7 Centre for Applied One Health Research and Policy Advice (OHRP), City University of Hong Kong, Hong Kong SAR, China

* Corresponding author

Younjung Kim (younjung.kim@stats.ox.ac.uk)

This Supplementary Information includes:

Figures S1 to S12

Table S1 and S2

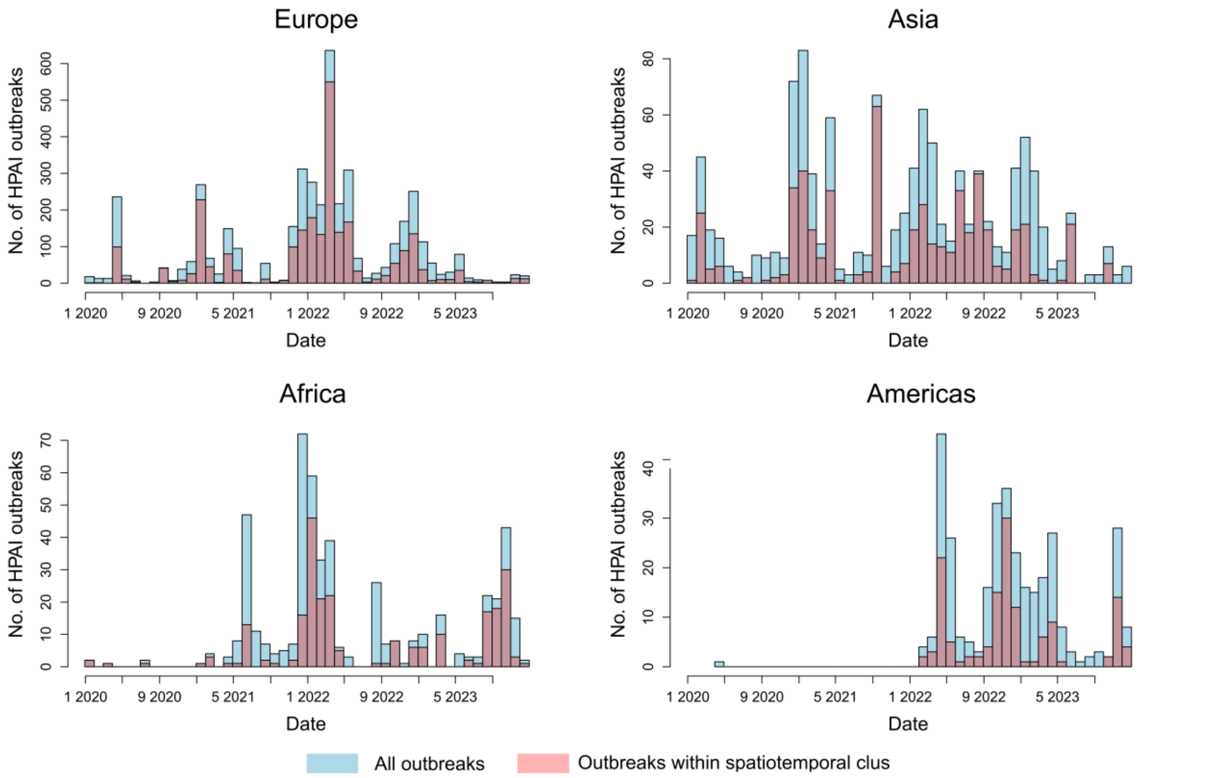

Figure S1 Distribution of HPAI outbreaks by region and spatiotemporal cluster. The bluish bars represent all analysed outbreaks, whereas the reddish bars indicate outbreaks classified as occurring within spatiotemporal clusters. Source data are provided as a Source Data file.

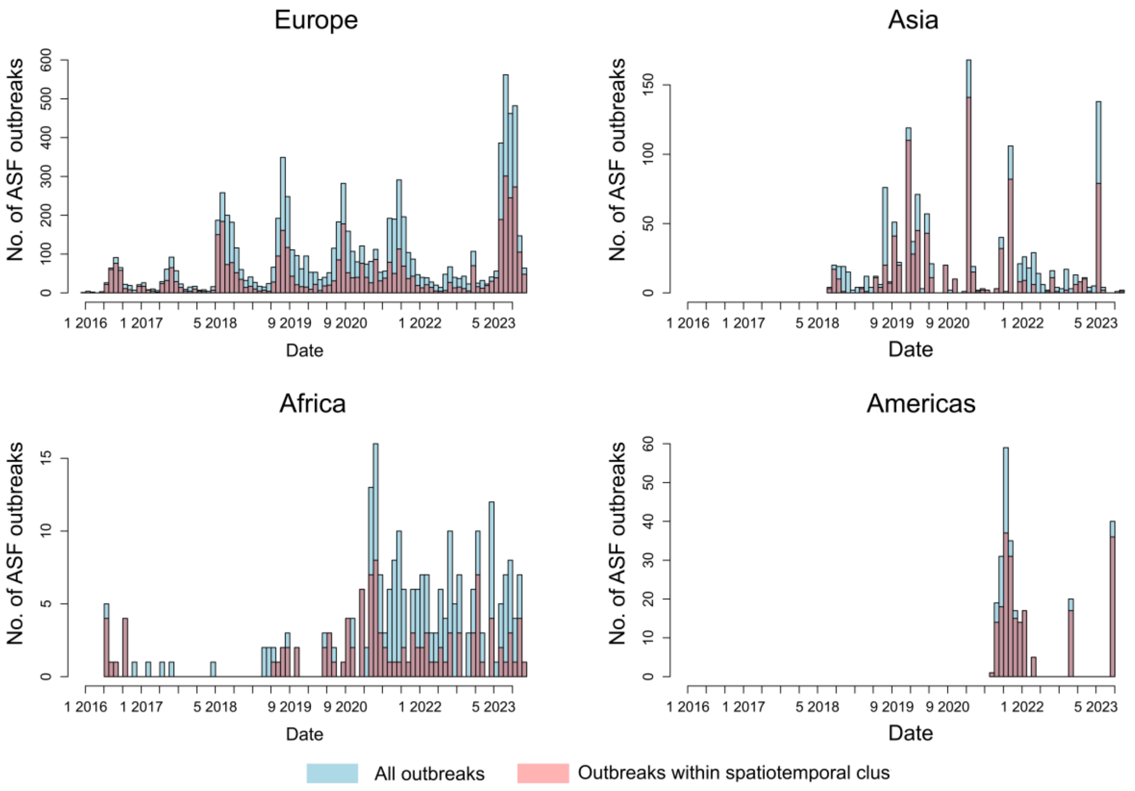

Figure S2 Distribution of ASF by region and spatiotemporal cluster. The bluish bars represent all analysed outbreaks, whereas the reddish bars indicate outbreaks classified as occurring within spatiotemporal clusters. Source data are provided as a Source Data file.

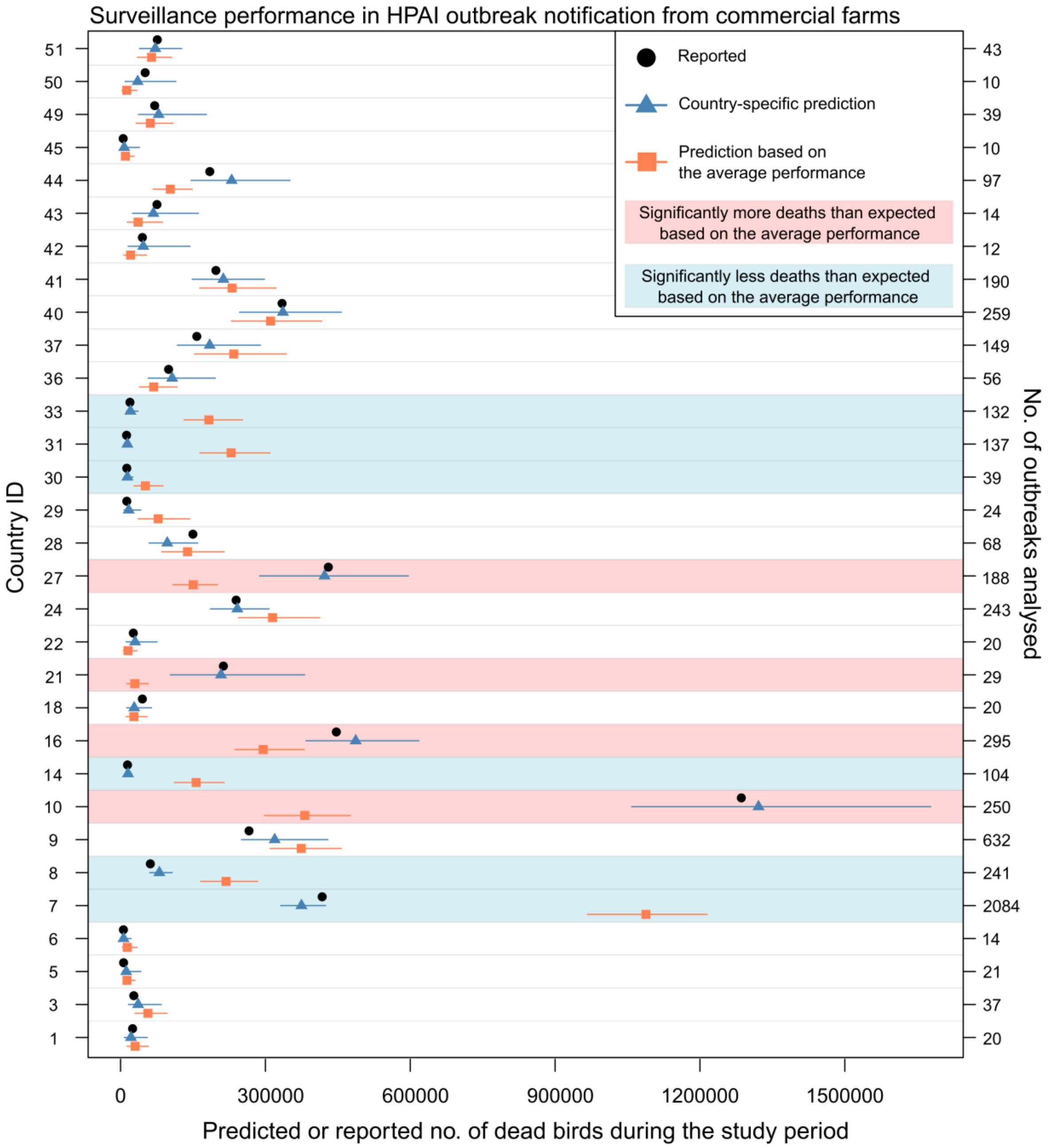

Figure S3 Country/territory-level performance in HPAI outbreak notification from commercial farms. The black points represent the total number of deaths across all analysed outbreaks (x-axis) by country/territory (y-axis), as recorded at initial notification. The blue triangles and horizontal lines represent the number of deaths predicted by the best-fitting model, while the orange squares and horizontal lines represent the number predicted by a model excluding the country/territory variable but including other variables from the best-fitting model. The blue background shading indicates countries estimated to have performed significantly better than average (i.e. the upper limit of the 95th percentile intervals [PI] predicted by the best-fitting model < lower limit of the 95th PI predicted by the model excluding country/territory). The red background shading indicates those estimated to have performed significantly worse than average (i.e. the lower limit of the 95th PI predicted by the best-fitting model > upper limit of the 95th PI predicted by the model excluding country/territory). Source data are provided as a Source Data file.

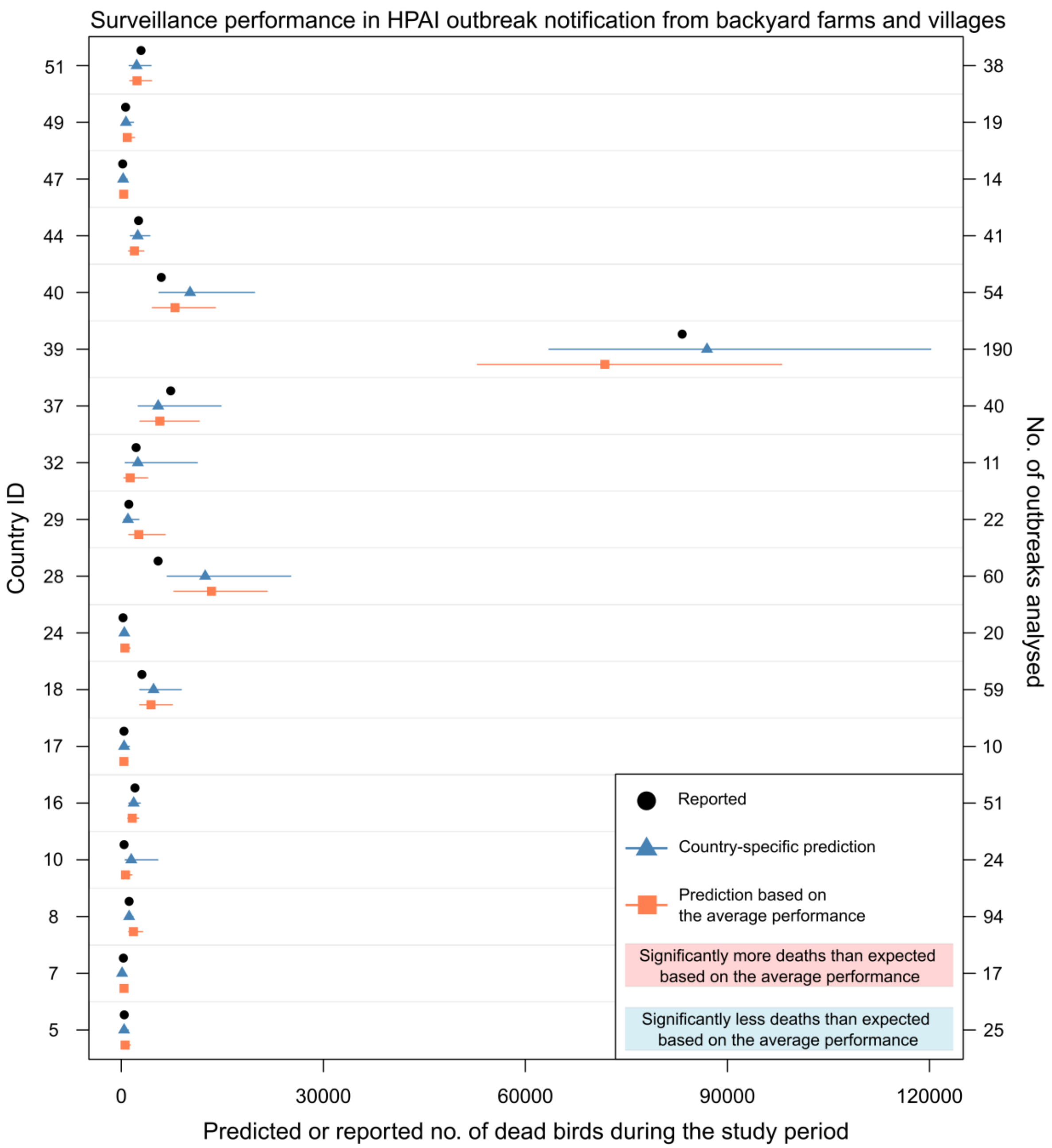

Figure S4 Country/territory-level performance in HPAI outbreak notification from backyard farms and villages. The black points represent the total number of deaths across all analysed outbreaks (x-axis) by country/territory (y-axis), as recorded at initial notification. The blue triangles and horizontal lines represent the number of deaths predicted by the best-fitting model, while the orange squares and horizontal lines represent the number predicted by a model excluding the country/territory variable but including other variables from the best-fitting model. The blue background shading indicates countries estimated to have performed significantly better than average (i.e. the upper limit of the 95th percentile intervals [PI] predicted by the best-fitting model < lower limit of the 95th PI predicted by the model excluding country/territory). The red background shading indicates those estimated to have performed significantly worse than average (i.e. the lower limit of the 95th PI predicted by the best-fitting model > upper limit of the 95th PI predicted by the model excluding country/territory). Source data are provided as a Source Data file.

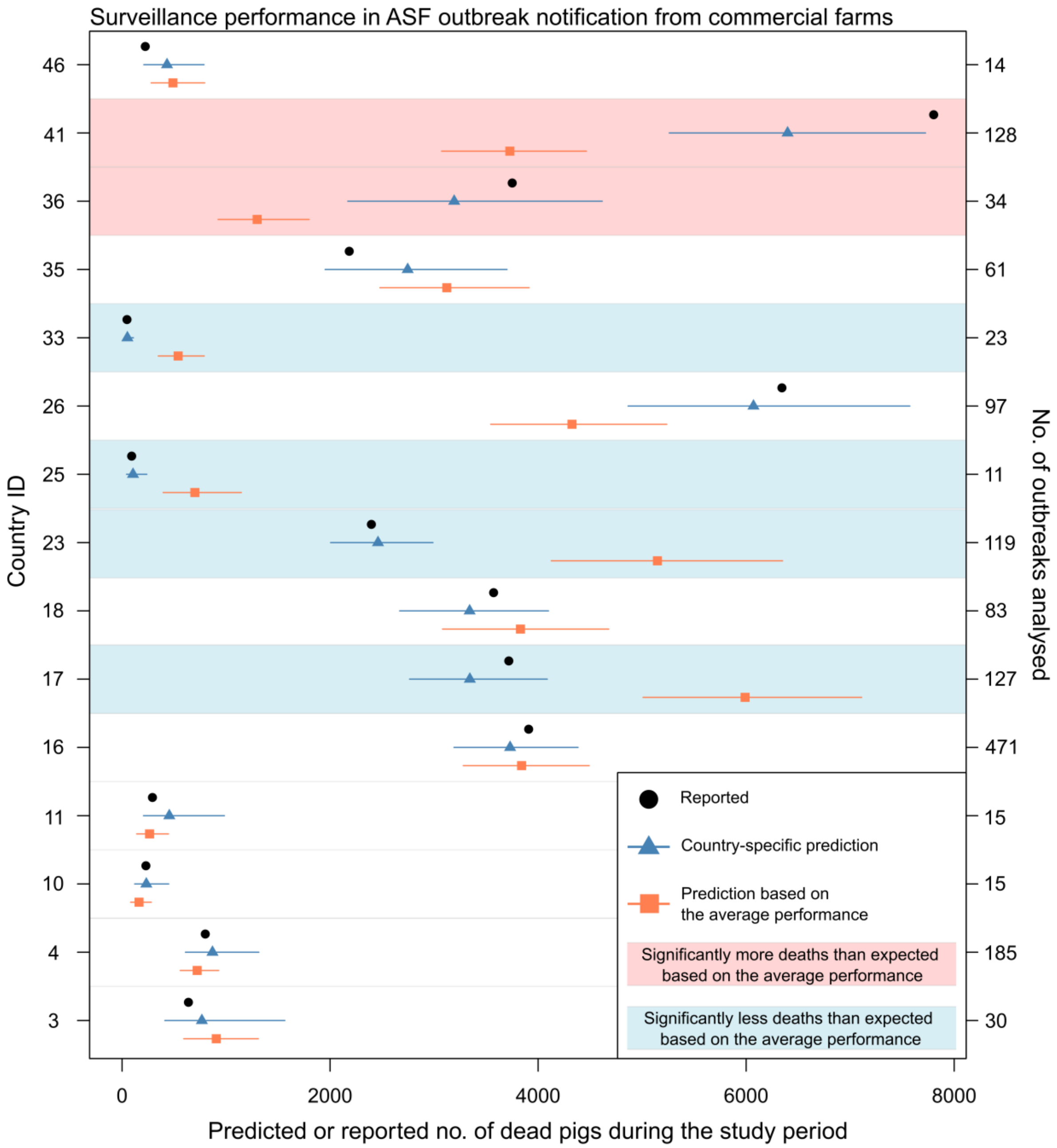

Figure S5 Country/territory-level performance in ASF outbreak notification from commercial farms. The black points represent the total number of deaths across all analysed outbreaks (x-axis) by country/territory (y-axis), as recorded at initial notification. The blue triangles and horizontal lines represent the number of deaths predicted by the best-fitting model, while the orange squares and horizontal lines represent the number predicted by a model excluding the country/territory variable but including other variables from the best-fitting model. The blue background shading indicates countries estimated to have performed significantly better than average (i.e. the upper limit of the 95th percentile intervals [PI] predicted by the best-fitting model < lower limit of the 95th PI predicted by the model excluding country/territory). The red background shading indicates those estimated to have performed significantly worse than average (i.e. the lower limit of the 95th PI predicted by the best-fitting model > upper limit of the 95th PI predicted by the model excluding country/territory). Source data are provided as a Source Data file.

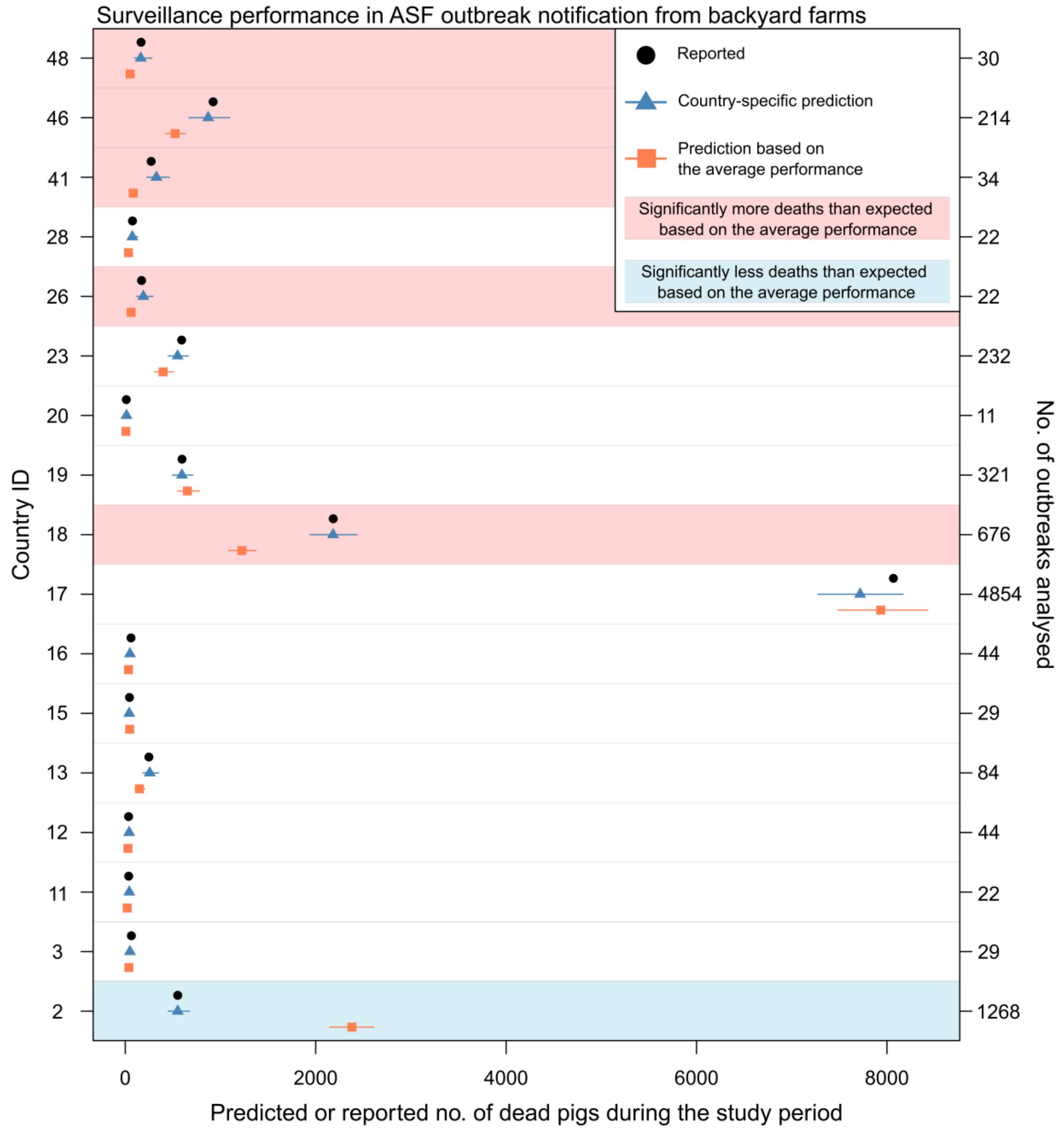

Figure S6 Country/territory-level performance in ASF outbreak notification from backyard farms. The black points represent the total number of deaths across all analysed outbreaks (x-axis) by country/territory (y-axis), as recorded at initial notification. The blue triangles and horizontal lines represent the number of deaths predicted by the best-fitting model, while the orange squares and horizontal lines represent the number predicted by a model excluding the country/territory variable but including other variables from the best-fitting model. The blue background shading indicates countries estimated to have performed significantly better than average (i.e. the upper limit of the 95th percentile intervals [PI] predicted by the best-fitting model < lower limit of the 95th PI predicted by the model excluding country/territory). The red background shading indicates those estimated to have performed significantly worse than average (i.e. the lower limit of the 95th PI predicted by the best-fitting model > upper limit of the 95th PI predicted by the model excluding country/territory). Source data are provided as a Source Data file.

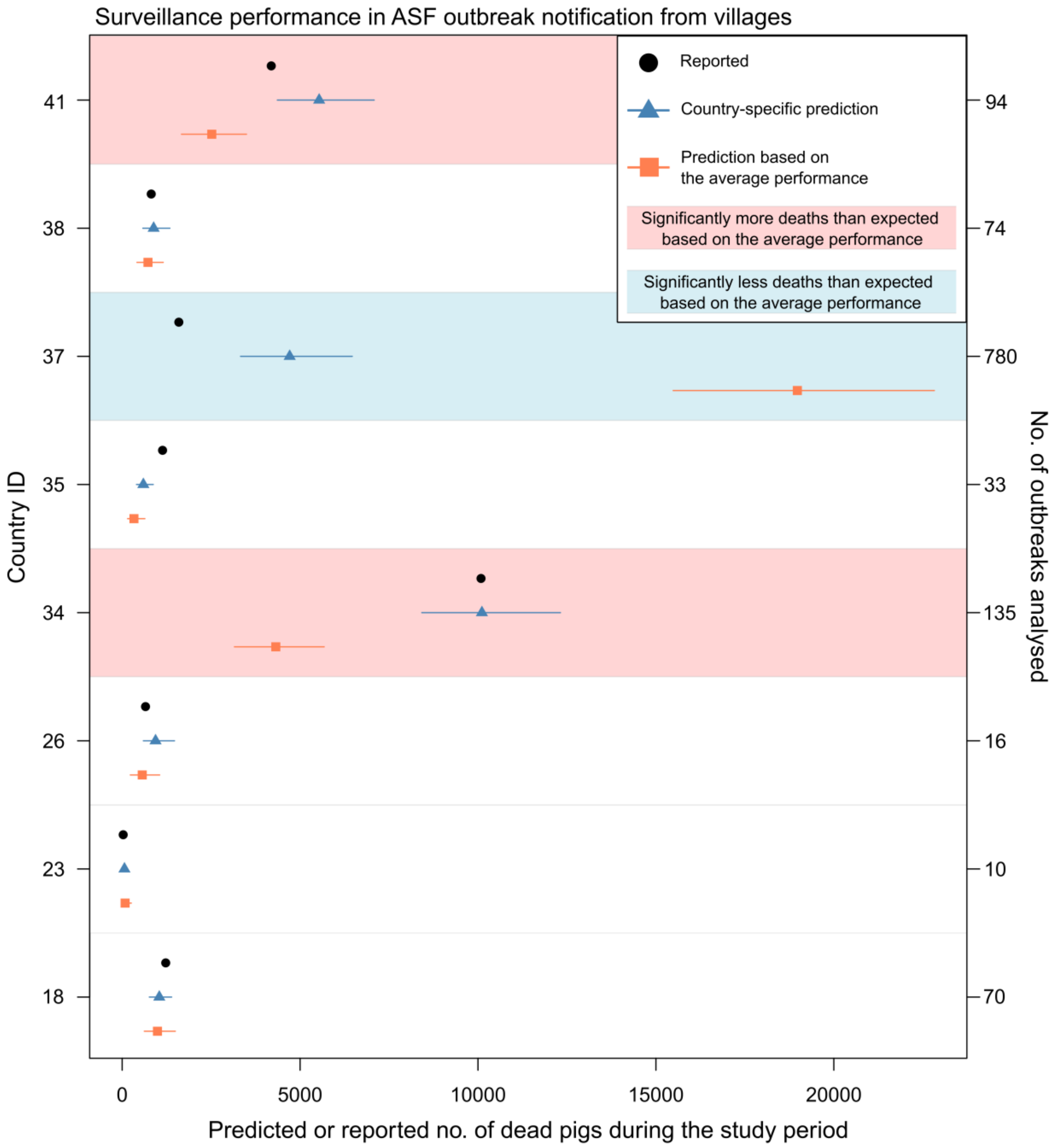

Figure S7 Country/territory-level performance in ASF outbreak notification from villages. The black points represent the total number of deaths across all analysed outbreaks (x-axis) by country/territory (y-axis), as recorded at initial notification. The blue triangles and horizontal lines represent the number of deaths predicted by the best-fitting model, while the orange squares and horizontal lines represent the number predicted by a model excluding the country/territory variable but including other variables from the best-fitting model. The blue background shading indicates countries estimated to have performed significantly better than average (i.e. the upper limit of the 95th percentile intervals [PI] predicted by the best-fitting model < lower limit of the 95th PI predicted by the model excluding country/territory). The red background shading indicates those estimated to have performed significantly worse than average (i.e. the lower limit of the 95th PI predicted by the best-fitting model > upper limit of the 95th PI predicted by the model excluding country/territory). Source data are provided as a Source Data file.

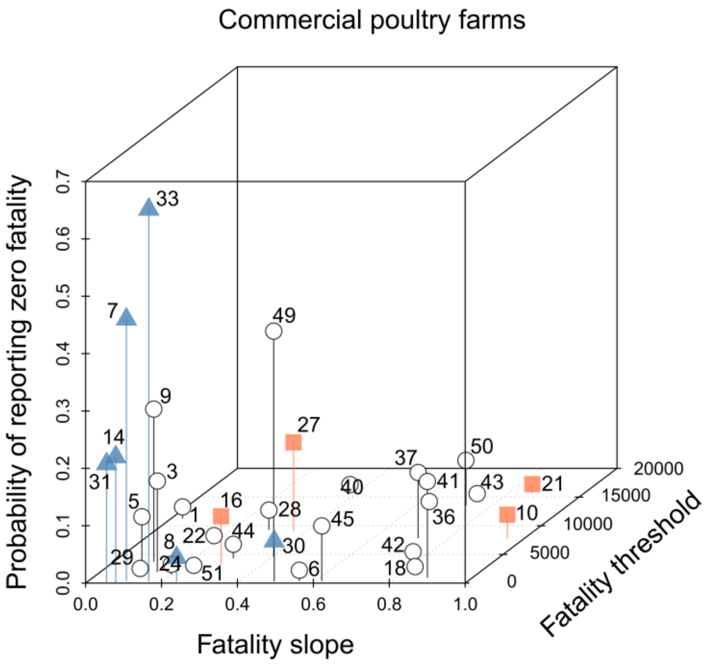

Figure S8 Country/territory-level fatality metrics for HPAI outbreaks on commercial farms. Each 3d dimensional plot shows the relationships between the three fatality metrics for HPAI outbreaks on commercial farms. The points represent countries/territories and are located according to the median posterior estimates of the fatality metrics estimated from the best-fitting models. The blue triangles represent the countries/territories estimated to have experienced significantly less deaths than expected from the average performance across all countries/territories, whereas the orange squares represent the countries/territories estimated to have experienced significantly more deaths than expected. These countries/territories are also highlighted in Figure S8. Source data are provided as a Source Data file.

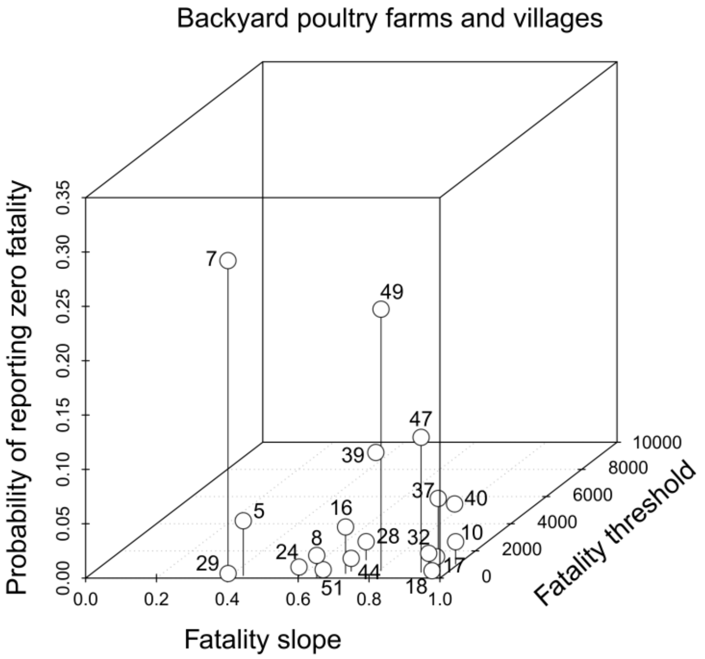

Figure S9 Country/territory-level fatality metrics for HPAI outbreaks on backyard farms and villages. Each 3d dimensional plot shows the relationships between the three fatality metrics for HPAI outbreaks on backyard farms and villages. The points represent countries/territories and are located according to the median posterior estimates of the fatality metrics estimated from the best-fitting models. Source data are provided as a Source Data file.

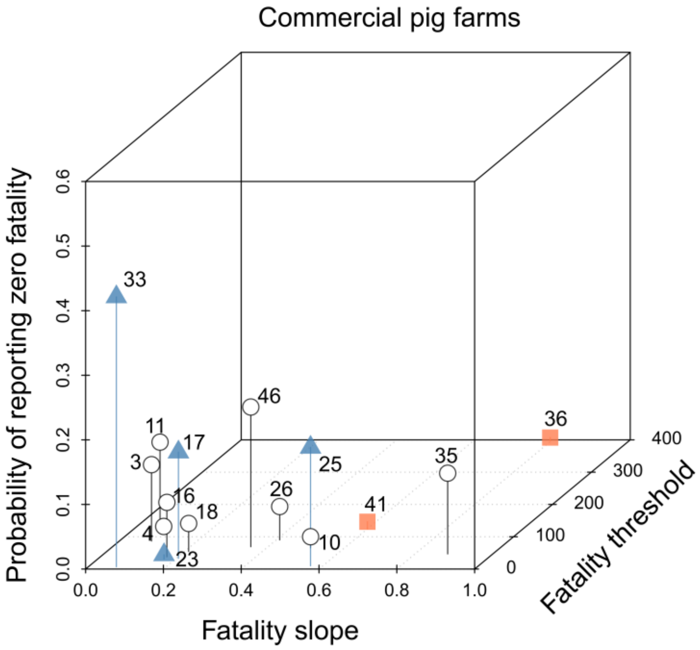

Figure S10 Country/territory-level fatality metrics for ASF outbreaks on commercial farms. Each 3d dimensional plot shows the relationships between the three fatality metrics for ASF outbreaks on commercial farms. The points represent countries/territories and are located according to the median posterior estimates of the fatality metrics estimated from the best-fitting models. The blue triangles represent the countries/territories estimated to have experienced significantly less deaths than expected from the average performance across all countries/territories, whereas the orange squares represent the countries/territories estimated to have experienced significantly more deaths than expected. These countries/territories are also highlighted in Figure S10. Source data are provided as a Source Data file.

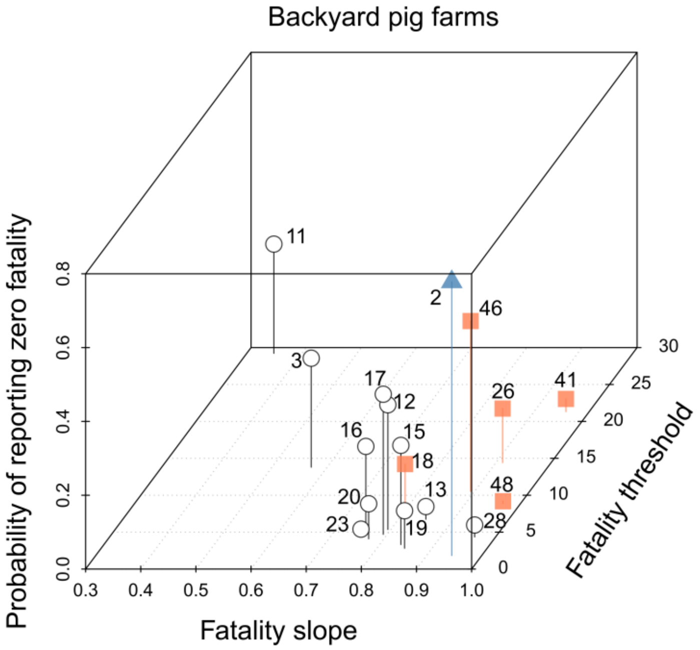

Figure S11 Country/territory-level fatality metrics for ASF outbreaks on backyard farms. Each 3d dimensional plot shows the relationships between the three fatality metrics for ASF outbreaks on backyard farms. The points represent countries/territories and are located according to the median posterior estimates of the fatality metrics estimated from the best-fitting models. The blue triangles represent the countries/territories estimated to have experienced significantly less deaths than expected from the average performance across all countries/territories, whereas the orange squares represent the countries/territories estimated to have experienced significantly more deaths than expected. These countries/territories are also highlighted in Figure S11. Source data are provided as a Source Data file.

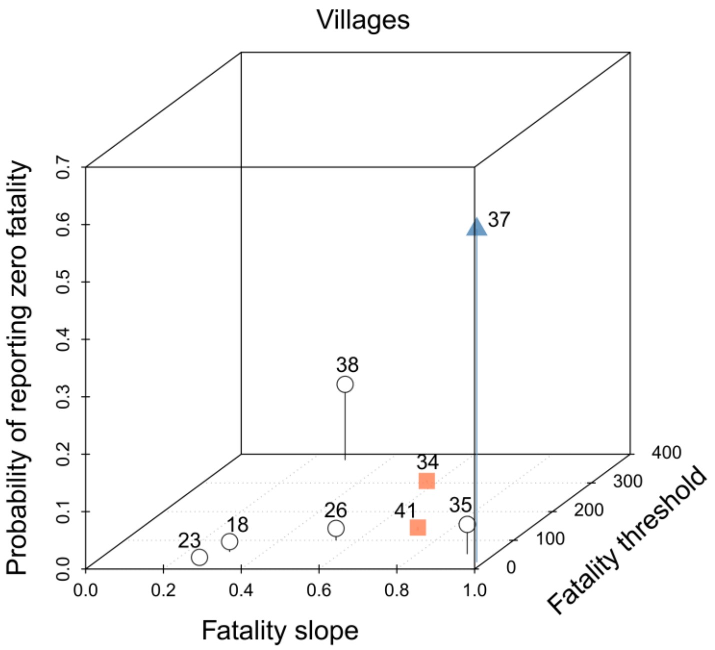

Figure S12 Country/territory-level fatality metrics for ASF outbreaks in villages. Each 3d dimensional plot shows the relationships between the three fatality metrics for ASF outbreaks in villages. The points represent countries/territories and are located according to the median posterior estimates of the fatality metrics estimated from the best-fitting models. The blue triangles represent the countries/territories estimated to have experienced significantly less deaths than expected from the average performance across all countries/territories, whereas the orange squares represent the countries/territories estimated to have experienced significantly more deaths than expected. These countries/territories are also highlighted in Figure S12. Source data are provided as a Source Data file.

Table S1 Proportion of HPAI outbreaks that had no information on the number of dead animals and premise size by country/territory

Country/ territory ID	Proportion of outbreaks with no information on the number of fatalities	Proportion of outbreaks with no information on the number of susceptible animals
51	0.735849057	0
6	0.322580645	0
33	0.32034632	0
3	0.173076923	0
42	0.111111111	0
30	0.066666667	0
7	0.060256978	0.005759858
9	0.035264484	0.022670025
37	0.032608696	0.097826087
41	0.012658228	0.042194093
44	0.008658009	0.008658009
16	0.00795756	0
10	0.005494505	0
27	0.005181347	0.005181347
1	0	0
43	0	0
45	0	0
5	0	0
47	0	0
8	0	0
28	0	0.006756757
29	0	0
31	0	0
32	0	0
49	0	0
36	0	0
14	0	0
40	0	0
50	0	0
17	0	0
18	0	0
21	0	0
22	0	0
24	0	0.003558719
39	0	0

Table S2 Proportion of ASF outbreaks that had no information on the number of dead animals and premise size by country/territory

Country/ territory ID	Proportion of outbreaks with no information on the number of fatalities	Proportion of outbreaks with no information on the number of susceptible animals
19	0.492331	0.001534
4	0.349315	0.003425
33	0.342105	0
37	0.277689	0.264045
38	0.217391	0.295652
2	0.089502	0
35	0.088496	0
28	0.021277	0
46	0.017241	0.013793
18	0.006322	0
17	0.001195	0.001991
25	0	0
3	0	0
26	0	0
48	0	0
10	0	0
34	0	0
11	0	0
12	0	0
13	0	0.03125
36	0	0
15	0	0
16	0	0
20	0	0
41	0	0.038062
23	0	0.046512
